# SCD and asthma comorbidity: the potential role of adverse childhood experiences

**DOI:** 10.1093/jscdis/yoaf007

**Published:** 2025-03-04

**Authors:** Brandi Pernell, Suzanne E. Perumean-Chaney, Teneasha Washington, Jessy Deshane, Elissa Epel, Alan Tita, Monica L. Baskin, Emily B. Levitan

**Affiliations:** 1 Division of Pediatric Hematology and Oncology, University of Alabama at Birmingham, Birmingham, AL 35233, United States; 2 School of Public Health, University of Alabama, Birmingham, AL 35294, United States; 3 Pulmonary, Allergy & Critical Care Medicine, University of Alabama at Birmingham, Birmingham, AL 35294, United States; 4 Department of Psychiatry, University of California, San Francisco, San Francisco, CA 94107, United States; 5 Obstetrics and Gynecology, University of Alabama at Birmingham, Birmingham, AL 35233, United States; 6 School of Medicine, University of Pittsburgh, Pittsburgh, PA 15261, United States

**Keywords:** SCD, asthma, Adverse Childhood Experiences (ACEs), early life stress, trauma, toxic stress

## Abstract

**Objectives:**

Asthma is strongly associated with poor health amongst individuals with SCD. Adverse Childhood Experiences (ACEs) are traumatic experiences occurring before 18 years of age. ACEs occur at the individual and familial (original ACEs) levels and expand to the community level (additional ACEs). Chronic exposure to ACEs leads to toxic stress, inflammation, and risk for chronic illnesses, including asthma. This study examined the relationship between ACEs and asthma among children and adolescents with SCD.

**Methods:**

Employing a cross-sectional study design, 75 children/adolescents with SCD were screened for ACEs. Prevalence ratios and logistic regressions were used to test the association and independent relationship between ACEs and asthma.

**Results:**

Fifty-nine (78%) participants reported exposure to at least 1 ACE. Adolescents exposed to ≥2 original- or ≥4 expanded ACEs (original + additional) were 1.15 times as likely to have asthma compared to those with no/low ACEs (95% CI: 1.03, 1.28, *P* = .01; 1.07, 1.24, *P* ≤ .001). Through logistic regression analyses, covarying age, sex, SCD genotype, income, and disease-modifying therapy, original (OR 1.52, 95% CI: 1.031, 2.242), additional (2.43, 95% CI: 1.335, 4.421), and expanded ACEs (1.529, 95% CI: 1.149, 2.036) were all shown to be independently associated with asthma.

**Conclusion:**

The findings from this research support our hypothesis that children/adolescents with SCD exposed to a higher number of ACEs have a higher prevalence of asthma compared to low-exposed subjects. This study lays the foundation for future longitudinal and interventional studies aimed to improve SCD-asthma outcomes through ACE-protective mechanisms.

## LAY SUMMARY

Sickle cell disease is an inherited disorder of the red blood cells (RBCs). RBCs supply oxygen throughout the body. Sickled RBCs not only change to an abnormal shape when exposed to oxygen-lowering triggers, but they also release harmful substances that incite widespread inflammation. Individuals with SCD are more likely to have asthma when compared to individuals without SCD, plausibly due to the inflammation inherent to SCD, and the consequent development of airway inflammation among a great majority of individuals with SCD. This predisposition for asthma is critical because individuals with SCD who also have asthma are at increased risk for SCD-related health complications, mainly pain and acute lung injury. Numerous research studies have demonstrated a connection between mental stress and asthma development but not in an SCD sample. In this study, 75 children and adolescents were surveyed for stress exposures. Study participants with a greater number of stress exposures were more likely to have asthma compared to participants with low stress exposures. This is the first SCD study to demonstrate the stress-asthma connection, and lays the foundation for future research aimed to prevent/buffer the impacts of stress on individuals with SCD and comorbid asthma.

## INTRODUCTION

Asthma is the most common chronic childhood condition, affecting an estimated 6.1 million children and adolescents across the globe.^1^ Asthma pathogenesis is multifactorial. Despite one’s genetic predispositions, an individual can develop asthma solely due to their environmental exposures.^2–4^ Common environmental exposures include smoking or secondhand smoke exposure, air pollutants, occupational irritants, and/or psychological stress. The recognition, management, and avoidance of asthma triggers are all critical, as undetected/uncontrolled asthma can lead to breathlessness, hypoxemia, potential hospitalization, dependence on transient bursts of systemic glucocorticoids, and even death. The consequences of undetected and/or uncontrolled asthma are particularly relevant for children with SCD, an inherited red blood cell disorder that is characterized by systemic inflammation, red blood cell hemolysis, vascular endothelial damage, and end-organ dysfunction/damage. Hypoxemia incites red blood cell polymerization/sickling, consequent red blood cell destruction and release of pro-inflammatory, pro-adhesive, and oxidative stress mediators, causing widespread inflammation, vascular endothelial damage, and end-organ dysfunction,^5^ including lung disease. This hypoxemia-induced pathology and resultant morbidity underscore the importance of early detection and effective management of asthma among individuals with SCD.

Numerous studies have documented the high prevalence of asthma among individuals with SCD in comparison to the general population. Specifically, according to the 2014 National Heart, Lung and Blood Institute SCD Guidelines, an estimated 35% of children with SCD are affected by asthma.^6^ This number is staggering in comparison to the reported 14% of African American children and 9% of children in general across the United States reported to have asthma. Longitudinal analyses have also demonstrated that, when compared to individuals who solely have SCD, individuals who have both SCD and asthma have higher incidence rates of vaso-occlusive pain and ACS,^7–11^ an acute, pneumonia-like illness that is documented to be the leading cause of critical care admission and death among children with SCD.^12^

Stress is well-connected to the pathogenesis of chronic health conditions by way of stress-induced programming of neuroendocrine, autonomic, immune, and inflammatory processes.^13^ Adverse Childhood Experiences (ACEs) are early life exposures to stress, occurring before 18 years of age. The landmark ACE study, led by Felitti et al.,^14^ highlighted the 10 original ACEs subcategorized as abuse, neglect, and household dysfunction. Through this pivotal research, Felitti et al.^14^ were able to demonstrate a dose-response relationship between ACEs and future development of chronic diseases, such as asthma. Fast forward 2 decades, not only has the original ACE measure been expanded to capture community-level stressors more likely to impact individuals of varying socioeconomic groups, such as bullying, experiences of racism and discrimination, neighborhood violence, and intimate partner violence, the ACE-asthma connection is now an internationally established phenomenon in the published literature. Findings from some of the largest published cohort studies have demonstrated a higher odds of asthma amongst subjects with multiple ACE exposures, typically in a dose-response fashion.^15–23^ One study examining the relationship between ACEs and SCD health has also been documented. In a previous study from our group, we found a higher frequency of hospitalizations for ACS among adolescents with SCD that had encountered ACEs compared to those with no reported ACE exposures.^24^ Among this same cohort, cumulative ACE scores were also found to be positively correlated with pain among adolescents with SCD.^24^ Considering the independent associations ACS, SCD pain, and ACEs all have with asthma, coupled with the disproportionate prevalence of asthma impacting the SCD population, it is critical to explore the ACE-asthma connection as a future potential target for improved SCD health outcomes.

We sought to examine the relationship between ACEs and asthma among children and adolescents with SCD by testing the hypothesis that children and adolescents with SCD with higher numbers of ACE exposures will have a higher prevalence of asthma, compared to those with no or a low number of ACE exposures. Given the high prevalence and risk for poor outcomes among individuals with SCD plus asthma, establishing this connection will lay the groundwork for future studies targeting protective/preventive ACE measures as a mitigating means to improved health outcomes among populations at higher risk of high healthcare utilization.

## MATERIALS AND METHODS

### Study design and participants

This cross-sectional study took place at the Comprehensive Pediatric Sickle Cell Center located at the University of Alabama at Birmingham and was approved by the local institutional review board. Sixty-eight percent of participant recruitment took place during routine outpatient follow-up encounters in the SCD-asthma clinic. The remaining subjects were approached for study participation during general outpatient SCD health maintenance encounters. Children and adolescents managed in the SCD-asthma clinic receive routine screening for asthma and asthma symptom control, age-appropriate pulmonary function testing, step-wise diagnosis and management according to the latest National Asthma Education and Prevention Program guidelines, written asthma action plans, and ongoing education and coaching regarding proper medication delivery technique. The study included male and female children and adolescents with confirmed SCD. All SCD genotypes were included in this study. Participants ranged in age from 1 to 19 years of age and were categorized as either children, 0–12 years of age, or adolescents/young adults, 13–19 years of age. Caregivers and participants 18 years of age and older provided consent. Participants 13–17 years of age provided assent.

### Data collection

#### Adverse childhood experiences

We followed the same ACE screening method as previous,^24^ employing the Center for Youth Wellness Adverse Childhood Experience Questionnaire (CYW ACE-Q), child and teen versions, which captures ACEs encountered by children and adolescents anytime from birth to present. The ACE exposures on the CYW ACE-Q are grouped into 2 sections. Section 1 includes the original ACEs, which capture experiences of parental/guardian separation/divorce, parental/guardian incarceration, poverty, physical or sexual abuse, neglect, and witnessing in-home domestic violence or substance abuse. Section 2 of the CYW ACE-Q expands on the original ACEs, capturing experiences of racism/discrimination, neighborhood violence, domestic partner violence, parental/guardian death, child-parent separation through immigration/deportation, incarceration, and having a life-threatening illness. The CYW ACE-Q tools were provided to participants 13 years of age and older and caregivers of children and adolescents 1–19 years of age for self- and parental completion. The original and additional ACEs were categorized as 0–1 vs ≥2 exposures and analyzed separately, as well as together (expanded ACEs). The expanded ACEs were categorized as 0–3 vs ≥4 exposures. These grouping methods were selected based on previous demonstrations in the literature of ≥2 ACEs being associated with SCD mice pathology^25^ and ≥4 ACEs being associated with chronic lung disease development.^14^ For scores that were incongruent between the adolescent and caregiver, we used the higher of the 2 scores.

#### Asthma definition

Our primary outcome of interest was a confirmed diagnosis of asthma. Asthma was operationalized as a binary (yes/no) variable. Study participants were categorized as having asthma if they or their caregiver had ever been told by an advanced care provider that they have asthma and/or had documentation of an asthma diagnosis in the electronic medical record system, plus active asthma medication prescriptions.

#### Baseline characteristics

We also captured disease-modifying therapy; zip codes, as a proxy for median household income; age and sex. These variables were examined at baseline amongst the ACE comparison groups to determine any statistically significant differences that may potentially influence our results.

### Statistical analysis

Study participant characteristics were summarized using descriptive statistics, with continuous variables being reported as means ± SD and categorical variables being reported as frequencies and percentages. These descriptive variables were compared among child and adolescent ACE exposure groups (0–1 vs ≥2 original and additional ACEs, and 0–3 vs ≥4 expanded ACEs; see “Materials and methods” and “Data Collection” section for explanation of categories) using a Wilcoxon rank-sum test and the chi-square (Fisher’s exact) test. Prevalence ratios were calculated employing Poisson regression analyses with robust standard errors to test the association between ACEs and asthma.^26^ A logistic regression was employed to examine the independent relationship between ACE exposures and asthma, controlling for relevant covariates. A *P*-value of <.05 was considered statistically significant employing two-tailed tests. All statistical analyses were performed using IBM SPSS Statistics 27 for Windows (IBM Corp, Armonk, NY, USA).

## RESULTS

### Demographics

Seventy-five children/adolescents 1–19 years of age were screened for ACEs during outpatient follow-up encounters using the CYW ACE-Q. All participants were African American. The mean age for this sample was 11.3 ± 4.8 years, with 53% identifying as male. Sixty percent of the study participants were younger children, ≤12 years of age (Table 1).

Examinations for statistically significant differences in participant characteristics were conducted amongst the child and adolescent groups exposed to 0–1 vs ≥2 original ACEs, 0–1 vs ≥2 additional ACEs and 0–3 vs ≥4 expanded ACEs. There was no detection of statistically significant difference in age, median household income, or disease-modifying therapies (hydroxyurea and/or chronic blood transfusion therapy) amongst any of the ACE comparison groups. The differences in sex distributions among the original and expanded ACE groups were statistically significant, in that greater than 80% of the male participants and/or their caregivers reported low exposures to ACEs, whereas female reports were more evenly distributed across the low vs high exposure groups (Table 2).

### Adverse childhood experiences

The average number of ACEs amongst the entire cohort was 2.52 ± 2.36. Parental separation/divorce was the most commonly reported ACE, followed by having a serious medical procedure or life-threatening illness and experiences of bullying at school. Among the child group, 31% reported 2 or more original ACEs, 29% reported 2 or more additional ACEs, and 33% reported 4 or more expanded ACEs. For the adolescent group, 30% reported 2 or more original ACEs and 33% reported 2 or more additional- and 4 or more expanded ACEs.

### Asthma

Among the 75 study participants, 45 (60%) had a confirmed comorbid diagnosis of asthma (Table 1). The differences in baseline characteristics among ACE comparison groups are displayed in Table 2. The distributions of asthma among the ACE comparison groups are displayed in Table 3. By age category, 50% (15 out of 30) of adolescents and 66% (30 out of 45) of the child participants had SCD + asthma (Table 4). The distribution of asthma was significantly higher amongst adolescents exposed to higher numbers of original, additional, and expanded ACEs (Table 4). Our prevalence ratios displayed in Table 5 demonstrated that, while holding age, SCD genotype, area-level income, disease-modifying therapy, and sex constant, children and adolescents with SCD exposed to ≥2 original or ≥4 expanded ACEs were 15% more likely to have a comorbid diagnosis of asthma compared to those with no/low ACEs (95% CI: 1.03, 1.28, *P* = .01; 95% CI: 1.07, 1.24, *P* ≤ .001, respectively). Children and adolescents with SCD exposed to ≥2 additional ACEs were 33% more likely to have a comorbid diagnosis of asthma compared to those with no/low additional ACEs (95% CI: 1.16, 1.51, *P* ≤ .001). Our logistic regressions demonstrated that, while holding age, SCD genotype, area-level income, disease-modifying therapy, and sex constant, the odds of having an asthma diagnosis increased by 52% (95% CI: 1.03, 2.24), 143% (95% CI: 1.34, 4.42); and 53% (95% CI: 1.15, 2.04) for every 1 unit increase in original, additional, or expanded ACE exposures, respectively (Table 6). Our Hosmer and Lemeshow tests were non-significant, indicating a good fit for all 3 logistic regression models. Our pseudo *R*^2^ values indicate that our current logistic regression models show a weak association among the predictors and asthma amongst the SCD-ACE exposure groups.

## DISCUSSION

Adverse Childhood Experiences are strongly associated with poor health outcomes across the lifespan. Several general population studies have specifically noted an increased risk for the development and exacerbation of asthma in connection to ACE exposures.^3,14,16^ This study marks the first examination of the ACE-asthma connection among children and adolescents with SCD, which is a critical unanswered question, given the disproportionately high prevalence of asthma and associated risk for increased morbidity and mortality among this high-risk population. The findings from this study support our a priori hypothesis that the prevalence of asthma would be higher among high ACE-exposed versus low-ACE-exposed children and adolescents with SCD.

The distribution of ACEs among our study sample mirrors distributions captured from general population studies among youth. The existing body of SCD literature highlights the relationship between acute psychological stress and acute pain among individuals with SCD. This study further adds to this growing area of research by demonstrating a potential connection between cumulative, early life stress and chronic disease development, specifically asthma. The benefits of ACE screening, in particular among children and adolescents with SCD, using the CYW ACE-Q, are that this tool enables the capture of not only parent-reported stressors but also child/adolescent reports as well. This differs from existing stress screening tools currently being utilized in the SCD community and may potentially allow for more discoveries of stress exposures, especially child-reported stressors that parents are oblivious to. The second most commonly reported ACE, having a life-threatening procedure or illness, was captured through the expanded section of the CYW ACE-Q. This ACE in particular deserves further exploration given its high frequency amongst this cohort to ascertain whether disease severity, catastrophizing, or other to-be-discovered factors correlate with perceptions of life-threatening status.

Our Fisher’s exact (original ACEs comparison groups) and Chi-square (additional and expanded ACEs comparison groups) analyses demonstrated a statistically significant difference in the distribution of asthma among the adolescent ACE exposure groups (Table 4). Given the peak incidence of ACS among children 2–4 years of age with SCD and the strong associations between ACS and asthma amongst individuals with SCD, as well as the asthma-ACE association amongst the general population, we anticipated a statistically significant difference in the distribution of asthma among high ACE-exposed vs low-exposed children with SCD. While this difference was not demonstrated among our child comparison groups, it is possible that with a larger sample size, we would have captured a statistically significant difference in asthma prevalence among the child comparison groups as well. The results from our prevalence ratios and logistic regressions indicate that a relationship does exist between original, additional, and expanded ACEs and asthma among children and adolescents with SCD adjusting for potential confounders, such as age, sex, area-level income, disease-modifying therapy, and SCD genotype. While our regression models were statistically significant, our pseudo *R*^2^ values indicate that other predictors not accounted for may be more strongly associated with the prevalence of asthma. These explanatory gaps support ongoing investigation of the connections between ACEs and asthma in SCD.

This study has several limitations worth mentioning. First, the external validity of our study findings may be called into question, as our sample had a prevalence of asthma almost twice the prevalence reported for pediatric SCD cohorts in the existing SCD literature. This higher prevalence of asthma is likely attributed to selection bias, as 68% of our study was recruited from an outpatient SCD-asthma and ACS follow-up clinic, both of which employ routine asthma screening as part of their standards of care. Conversely, there is also the potential for an underrepresentation of asthma among this sample, both due to missing asthma diagnoses documented in the medical record as well as a delay in asthma diagnoses due to some clinician’s hesitancy to diagnose a child with asthma until age 5 or 6 years or greater, the age which most children are able to start performing spirometry. Our use of a cross-sectional study design prevented the establishment of a temporal relationship between our exposure and outcome of interest. Our ACE measure captured the type of exposures but not the timing of each ACE exposure, further adding to the issue of temporality. A true understanding of the relationship between 2 variables requires consideration of potential confounders. Potential confounders this study failed to capture are second-hand smoke exposure; parental and/or sibling history of asthma; exposure and duration of exclusive breastfeeding; neighborhood air quality indices, proximity to industrial plants, and other sources of air pollution; and body mass index. Also, household income per capita is a more informative representation of socioeconomic status, as opposed to area-level income, and will be employed with future prospective studies associated with this line of research. There is also the potential for social desirability/reporting bias, given the sensitivity of certain ACEs and reluctance to disclose them. Given that >80% of the male participants reported low ACE exposures, it is possible that either the CYW ACE-Q does not fully capture the specific exposures encountered by males with SCD or the males that participated in this study were hesitant to fully disclose the stressors they’ve encountered. Also, given the strong likelihood that individuals with more severe SCD genotypes are more likely to experience complex, adverse, and/or potentially life-threatening conditions, future studies should account for the mediating effect of disease severity on the relationship between ACE exposures and asthma. Lastly, our limited sample size prohibited our ability to control for relevant confounding variables.

## CONCLUSION

Asthma was found to be more prevalent among children and adolescents with SCD exposed to ACEs compared to participants with SCD who had not been exposed to ACEs. Capturing the expanded ACEs helped identify several frequently encountered stressors amongst child and adolescent participants. These findings lay the groundwork for future studies focused on ACEs and asthma in SCD. Specifically, future prospective studies are needed to further examine the relationship between ACEs and asthma, continue examining the utility of expanded ACE measures such as the CYW-Q, establish temporality, and examine the feasibility of ACE-preventive/protective interventions among children and adolescents with SCD.

## Figures and Tables

**Table 1.** Descriptive statistics of entire cohort.

Patient characteristics, *n* (%)	All (*n*=75)
Gender	
Male	40 (53.3)
Female	35 (46.7)
Age (years), mean ± SD	11.3 ± 4.8
Age group	
Child	45 (60)
Adolescent	30 (40)
SCD phenotype	
Hgb SS/SBthal^0^	68 (90.7)
Hgb SC/SBthal^+^	7 (9.3)
Area level income ($), mean ± SD	41725.7 ± 15237.9
Disease-modifying therapy	
No	11 (14.7)
Yes	64 (85.3)
Original ACE	
0–1	52 (69.3)
≥2	23 (30.7)
Additional ACE	
0–1	52 (69.3)
≥2	23 (30.7)
Expanded ACE	
0–3	50 (66.7)
≥4	25 (33.3)
Asthma	
No	30 (40)
Yes	45 (60)

Abbreviation: ACE = Adverse Childhood Experience.

**Table 2.** Baseline characteristics by ACE category.

Original ACE			
Patient characteristics, *n* (%)	0–1 ACE (*n* = 52)	≥2 ACE (*n* = 23)	*P*-value
Age (years), mean ± SD	11.25 ± 4.5	11.35 ± 5.5	.995
Gender			.008^a^
Male	33 (63.5)	7 (30.5)	
Female	19 (36.5)	16 (69.5)	
Area-level income ($), mean ± SD	39 535 ± 13 616	46 679 ± 17 728	.090
Disease-modifying therapy			1.000^b^
No	8 (15.4)	3 (13)	
Yes	44 (84.6)	20 (87)	
Additional ACE			
Patient characteristics, *n* (%)	0–1 ACE (*n* = 52)	≥2 ACE (*n* = 23)	
Age (years), mean ± SD	11 ± 4.6	11.83 ± 5.2	.568
Gender			.101^a^
Male	31 (60)	9 (39)	
Female	21 (40)	14 (61)	
Area-level income ($), mean ± SD	41297 ± 14386	42694 ± 17316	.761
Disease-modifying therapy			.728^b^
No	7 (13.5)	4 (17.4)	
Yes	45 (86.5)	19 (82.6)	
Expanded ACE			
Patient characteristics, n(%)	0–3 ACE (n = 50)	>4 ACE (n = 25)	
Age (years), mean ± SD	11.28 ± 4.4	11.28 ± 5.5	.946
Gender			.033^a^
Male	31 (62)	9 (36)	
Female	19 (38)	16 (64)	
Area-level income ($), mean ± SD	40797 ± 14779	43583 ± 16268	.421
Disease-modifying therapy			.490^b^
No	6 (12)	5 (20)	
Yes	44 (88)	20 (80)	

Abbreviation: ACE = Adverse Childhood Experience.

a Chi-square.

b Fisher’s exact.

**Table 3.** Distribution of asthma by ACE category

Original ACE			
	0–1 ACE (*n* = 52)	≥2 ACE (*n* = 23)	*P*-value
Asthma, *n* (%)			.008
No	26 (50)	4 (17.4)	
Yes	26 (50)	19 (82.6)	
Additional ACE			
	0–1 ACE (*n* = 52)	≥2 ACE (*n* = 23)	
Asthma, *n* (%)			.008
No	26 (50)	4 (17.4)	
Yes	26 (50)	19 (82.6)	
Expanded ACE			
	0–3 ACE (*n* = 50)	≥4 ACE (*n* = 25)	
Asthma, *n* (%)			.012
No	25 (50)	5 (20)	
Yes	25 (50)	20 (80)	

Abbreviation: ACE = Adverse Childhood Experience.

Chi-square analyses.

**Table 4.** Distribution of asthma by age and ACE categorization group

Original ACEs						
	Child group (*n* = 45)		*P*	Adolescent group (*n* = 30)		*P*
	0–1 ACE (*n* = 31)	≥2 ACE (*n* = 14)		0–1 ACE (*n* = 21)	≥2 ACE (*n* = 9)	
Asthma, *n* (%)			0.321^b^			.014^b^
No	12 (38.7)	3 (21.4)		14 (66.7)	1 (11.1)	
Yes	19 (61.3)	11 (78.6)		7 (33.3)	8 (88.9)	
Additional ACE						
	Child group (*n* = 45)			Adolescent group (*n* = 30)		*P*
	0–1 ACE (*n* = 32)	≥2 ACE (*n* = 13)		0–1 ACE (*n* = 20)	≥2 ACE (*n* = 10)	
Asthma, *n* (%)			0.165^b^			.020^a^
No	13 (40.6)	2 (15.4)		13 (65)	2 (20)	
Yes	19 (59.4)	11 (84.6)		7 (35)	8 (80)	
Expanded ACE						
	Child group (*n* = 45)			Adolescent group (*n* = 30)		
	0–3 ACE (*n* = 30)	≥4 ACE (*n* = 15)		0–3 ACE (*n* = 20)	≥4 ACE (*n* = 10)	*P*
Asthma, *n* (%)			0.180^b^			.020^a^
No	12 (40)	3 (20)		13 (65)	2 (20)	
Yes	18 (60)	12 (80)		7 (35)	8(80)	

Abbreviation: ACE = Adverse Childhood Experience.

a Chi-square.

b Fisher’s exact.

**Table 5.** Poisson regressions with robust standard errors modeled to estimate prevalence ratios of asthma.

Original ACEs				
Variable	Reference group	Prevalence ratio	95% Confidence interval	*P*-value
Age		0.972	0.939, 1.006	.109
SCD genotype	SS			
SB^0^thal		0.747	0.403, 1.386	.356
SB^+^thal		1.064	0.475, 2.383	.880
SC		1.680	1.055, 2.677	.029
Area-level income		1.00	1.00, 1.00	.354
Disease-modifying therapy	No	1.232	0.677, 2.243	.495
Sex	Female	1.247	0.822, 1.891	.300
Original ACEs		1.151	1.034, 1.282	.010
Additional ACEs				
Variable	Reference group	Prevalence ratio	95% Confidence interval	*P*-value
Age		0.971	0.938, 1.006	.107
SCD genotype	SS			
SB^0^thal		0.846	0.439, 1.631	.618
SB^+^thal		0.826	0.327, 2.082	.685
SC		2.426	1.498, 3.930	<.001
Area-level income		1.0	1.0, 1.0	.390
Disease-modifying therapy	No	1.257	0.663, 2.382	.483
Sex	Female	1.265	0.862, 1.855	.229
Additional ACEs		1.325	1.163, 1.510	<.001
Expanded ACEs				
Variable	Reference group	Prevalence ratio	95% Confidence interval	*P*-value
Age		0.971	0.939, 1.003	.076
SCD genotype	SS			
SB^0^thal		0.747	0.403, 1.386	.356
SB^+^thal		0.876	0.364, 2.107	.768
SC		1.861	1.153, 3.002	.011
Area-level income		1.0	1.0, 1.0	.375
Disease-modifying therapy	No	1.239	0.693, 2.218	.470
Sex	Female	1.367	0.898, 2.082	.145
Expanded ACEs		1.148	1.067, 1.236	<.001

Abbreviation: ACE = Adverse Childhood Experience.

**Table 6.** Logistic regressions modeled to predict the odds of asthma.

Original ACEs				
Variable	Reference group	Odds ratio	95% Confidence interval	*P*-value
Age		0.920	0.824, 1.028	.143
SCD genotype	SS	1.466	0.579, 3.710	.420
Area-level income		1.00	1.00, 1.00	.175
Disease-modifying therapy	Yes	0.469	0.093, 2.355	.358
Sex	Male	0.592	0.200, 1.749	
Original ACEs		1.521	1.031, 2.242	.034
Additional ACEs				
Variable	Reference group	Odds ratio	95% Confidence interval	*P*-value
Age		0.913	0.814, 1.024	.121
SCD genotype	SS	1.411	0.590, 3.375	.440
Area-level income		1.0	1.0, 1.0	.191
Disease-modifying therapy	Yes	0.476	0.091, 2.476	.377
Sex	Male	0.472	0.150, 1.488	.200
Additional ACEs		2.430	1.335, 4.421	.004
Expanded ACEs				
Variable	Reference group	Odds ratio	95% Confidence interval	*P*-value
Age		0.910	0.809, 1.023	.115
SCD genotype	SS	1.328	0.516, 3.415	.557
Area-level income		1.0	1.0, 1.0	.163
Disease-modifying therapy	Yes	0.446	0.081, 2.451	.353
Sex	Male	0.426	0.132, 1.379	.155
Expanded ACEs		1.529	1.149, 2.036	.004

Hosmer and Lemeshow test: *P* = .856, Cox & Snell *R*^2^: 0.120.

Hosmer and Lemeshow test: *P* = .914, Cox and Snell *R*^2^: 0.189.

Hosmer and Lemeshow test: *P* = .534, Cox and Snell *R*^2^: 0.181.

## Data Availability

Data are available upon request to the corresponding author.
